# The influence of rosmarinic acid on male reproductive health: Biological mechanisms and clinical prospects

**DOI:** 10.1016/j.bbrep.2026.102610

**Published:** 2026-05-03

**Authors:** Morteza Jafarinia, Sepide Goharitaban, Bahia Namavar Jahromi, Mohammad Ebrahim Parsanezhad, Sina Vakili

**Affiliations:** a Shiraz Neuroscience Research Center, Shiraz University of Medical Sciences, Shiraz, Iran; b Department of Anatomical Sciences, Faculty of Medicine, Hamedan University of Medical Science, Hamedan, Iran; c Infertility Research Center, Shiraz University of Medical Sciences, Shiraz, Iran; d Department of Obstetrics and Gynecology, School of Medicine, Shiraz University of Medical Sciences, Shiraz, Iran

**Keywords:** Rosmarinic acid, Male reproductive health, Oxidative stress

## Abstract

Male infertility is a condition affecting up to 15% of couples worldwide. It is frequently driven by oxidative stress and inflammation that impair spermatogenesis and hormone regulation. Rosmarinic acid (RA) as a naturally occurring polyphenolic compound has appeared as a potent antioxidant and anti-inflammatory agent with promising reproductive benefits. According to experimental models, RA reduces testicular damage brought on by oxidative stress, restores antioxidant enzyme activity, maintains testosterone and gonadotropin levels, and preserves sperm motility and morphology. These effects are mechanistically mediated through inhibition of inflammatory cytokines, protection of Leydig cell steroidogenesis, modification of NF-κB and Nrf2 pathways, and radical scavenging. There is still a lack of sufficient human data despite substantial preclinical validation, and concerns about bioavailability and standardized dosage call for more research. This review consolidates current evidence on RA's chemistry, pharmacokinetics, and mechanistic actions in the male reproductive system.

## Highlights

• Rosmarinic acid protects testicular tissue from oxidative stress and inflammation.
• RA preserves sperm count, motility, morphology, and testicular architecture in models.
• RA maintains testosterone and gonadotropin levels under toxic or metabolic stress.
• Antioxidant and anti-inflammatory pathways (NF-κB, Nrf2) mediate RA's protective effects.
• Current evidence is preclinical; human trials are needed to confirm fertility benefits.

## 1 Introduction

Infertility is a major global health concern, affecting approximately 10–15% of couples, with male factors contributing to nearly half of these cases [1,2]. Recent meta-analyses indicate a marked decline in sperm counts, nearly 50% from 1973 to 2018, an alarming trend that appears to be accelerating in the 21st century [3]. Male infertility arises from a multifactorial etiology, including genetic defects, endocrine imbalances, varicocele, infections, and detrimental lifestyle choices [4]. Environmental toxicants such as pesticides, heavy metals, and industrial chemicals further compromise spermatogenesis and sperm function [5,6]. For instance, the widespread contaminant perfluorooctanoic acid (PFOA) can induce male infertility and germ cell DNA damage via oxidative stress [7]. Despite progress in assisted reproductive technologies, identifying and mitigating the root causes of male infertility remain formidable challenges. Oxidative stress (OS) is now recognized as a pivotal factor in male infertility [8]. Spermatozoa are particularly vulnerable to excess reactive oxygen species (ROS) because they possess limited intrinsic antioxidant defenses and are rich in polyunsaturated fatty acids. This imbalance can damage sperm lipids, proteins, and DNA, impairing count, motility, morphology, and genomic integrity [9]. Chronic inflammation often coexists with OS; infections or autoimmune responses in the male reproductive tract trigger cytokine release and leukocyte infiltration, further amplifying ROS generation [10]. Persistent inflammatory insults can disrupt the blood–testis barrier, hinder spermatogenesis, and promote germ cell apoptosis. Collectively, oxidative stress and inflammation form a unifying pathogenic axis in numerous causes of male infertility, highlighting the therapeutic potential of interventions targeting these pathways. Augmenting antioxidant capacity has shown promise in counteracting male infertility linked to OS [11]. While endogenous seminal plasma antioxidants offer partial protection, exogenous agents, including vitamins, carotenoids, and polyphenols, have been explored to bolster sperm quality [12]. Clinical trials examining dietary antioxidants (e.g., coenzyme Q10, carnitine, vitamin E) report improvements in sperm concentration, motility, and DNA integrity in selected populations [13,14]. Herbal remedies and phytochemicals are of particular interest, given their multitargeted bioactivities and long-standing use in traditional medicine [15]. Within this broad category, polyphenolic compounds have garnered attention for their potent free-radical scavenging and anti-inflammatory effects, with increasing evidence suggesting they may enhance male reproductive outcomes. Rosmarinic acid (RA), a polyphenolic ester of caffeic acid and 3,4-dihydroxyphenyllactic acid, is abundant in culinary and medicinal herbs of the Lamiaceae family (e.g., rosemary, sage, peppermint) [16]. Its multiple hydroxyl groups confer robust antioxidant activity, and it also exhibits anti-inflammatory, antiviral, antibacterial, neuroprotective, and antitumor properties [17]. Notably, RA demonstrates low toxicity, making it an attractive nutraceutical candidate. Emerging data suggest that RA can mitigate oxidative damage in sperm and testicular tissues, thereby preserving male fertility [18,19]. This review provides a comprehensive overview of RA's chemistry, sources, and mechanisms of action in the context of male infertility, along with experimental evidence supporting its protective effects on sperm quality and testicular function. Safety considerations, formulation strategies, and future prospects for clinical application are also discussed.

## 2 Chemistry and sources of rosmarinic acid

### 2.1 Chemical structure and antioxidant properties

**Fig. 1:**
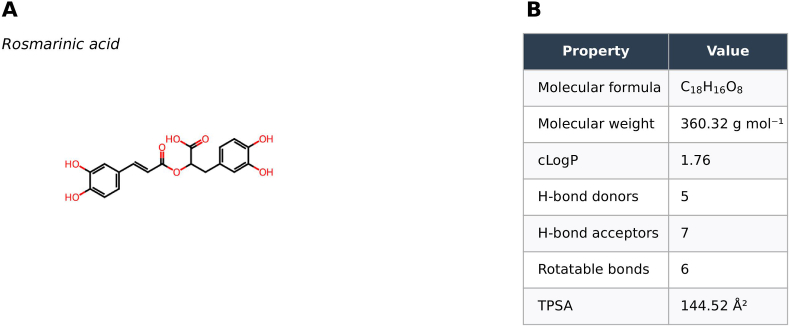
Structural characterization of rosmarinic acid (RA). (A) 2D representation with atom indices highlighting molecular connectivity. (B) Key molecular properties, including formula, molecular weight, logP, H-bond donors, H-bond acceptors, and rotatable bonds, providing physicochemical insights.

RA is a polyphenolic ester (C18H16O8) comprising a caffeic acid moiety linked to 3,4-dihydroxyphenyllactic acid, resulting in four aromatic hydroxyl groups and multiple conjugated double bonds [16,20] (Fig. 1A). Key physicochemical features relevant to absorption and distribution are summarized separately (Fig. 1B). These structural features allow effective resonance stabilization of free radicals, underlining its strong antioxidant activity. RA donates hydrogen atoms or electrons to neutralize reactive species, forming resonance-stabilized phenoxy radicals that interrupt lipid peroxidation chain reactions [21]. In chemical assays, RA frequently surpasses standard antioxidants, such as Trolox, in scavenging radicals, including the 1,1-diphenyl-2-picrylhydrazyl (DPPH)-2,2-diphenyl-1-picrylhydrazyl (DPPH) radical [22]. Its phenolic hydroxyl groups also enable metal chelation, thereby quenching redox-active metal ions and reactive species like superoxide anion, hydroxyl radical, singlet oxygen, and peroxynitrite [22]. Beyond direct radical scavenging, RA can enhance endogenous antioxidant defenses; studies have shown upregulation of antioxidant enzymes such as superoxide dismutase, catalase, and glutathione peroxidase in tissues under oxidative stress [23,24]. Furthermore, its conjugated structure can absorb ultraviolet (UV) radiation, mitigating UV-induced oxidative damage. Overall, these physicochemical properties position RA as a potent defender against oxidative injury in biological systems.

### 2.2 Natural occurrence and sources

RA is abundant in plants from the Lamiaceae (mint) and Boraginaceae families, most notably in rosemary (Rosmarinus officinalis), sage (*Salvia officinalis*), thyme (*Thymus vulgaris*), oregano (*Origanum vulgare*), perilla (*Perilla frutescens*), lemon balm (*Melissa officinalis*), basil (*Ocimum basilicum*), peppermint (*Mentha piperita*), and bugleweed (*Prunella vulgaris*) [25,26]. Reported RA concentrations vary substantially by species, cultivar, harvest season, and extraction conditions; Table 1 summarizes representative RA contents in common culinary/medicinal Lamiaceae herbs to provide a practical reference point [27]. In many of these herbs, RA constitutes a major phenolic component; for instance, optimized extraction protocols from dried rosemary leaves can yield approximately 0.34% RA by weight [28]. Seasonal variations, soil conditions, and cultivation practices influence RA levels, with basil plants showing particularly wide fluctuations in RA content [29]. Its prevalence in commonly consumed herbs has supported its long history of dietary and medicinal use, underscoring its status as a nutraceutical compound with an established safety record.

**Table 1:** Natural sources of rosmarinic acid (RA) and representative contents in common Lamiaceae herbs. Values are reported as mean ± SD in ethanolic extracts; content can vary with cultivar, harvest, and extraction conditions.

Plant (common name)	Species (family)	Extract matrix (as reported)	RA content (mg/g extract)	Notes/practical interpretation	Reference
Oregano	*Origanum vulgare* (Lamiaceae)	Ethanolic extract	12.40 ± 0.08	Among commonly used culinary herbs, oregano is often RA-rich under ethanolic extraction conditions.	[27]
Lemon balm	*Melissa officinalis* (Lamiaceae)	Ethanolic extract	7.84 ± 0.07	A frequent source for standardized RA-rich preparations used in human PK/clinical studies.	[27]
Basil	*Ocimum basilicum* (Lamiaceae)	Ethanolic extract	3.59 ± 0.01	RA content is variable across cultivars and growth conditions; values shown are representative.	[27]
Hyssop	*Hyssopus officinalis* (Lamiaceae)	Ethanolic extract	2.85 ± 0.004	Moderate RA content in ethanolic extracts.	[27]
Sage	*Salvia officinalis* (Lamiaceae)	Ethanolic extract	2.12 ± 0.02	Moderate RA content; widely used medicinal herb.	[27]
Rosemary	Rosmarinus officinalis (Lamiaceae)	Ethanolic extract	1.33 ± 0.01	Reported extract content varies with plant material and extraction; optimized workflows can increase recoverable RA.	[27]

### 2.3 Extraction and purification

RA is commonly extracted from plant materials using polar solvents such as methanol, ethanol, water, or acetone [30]. Extraction efficiency depends on factors including solvent composition, temperature, extraction time, and plant particle size. Advanced techniques, such as ultrasound- or microwave-assisted extraction and supercritical fluid extraction, have been developed to improve yields and preserve RA's stability [31]. Subsequent purification typically involves chromatographic methods (e.g., reversed-phase HPLC) to separate RA from other phenolics and flavonoids [32]. Alternative production routes include plant tissue culture systems and engineered microbial fermentation, leveraging the compound's biosynthetic pathway through caffeic acid and tyrosine-derived dihydroxyphenyllactic acid [33,34]. Purified RA is generally water-soluble and appears as a beige or brownish powder with a characteristic UV absorbance maximum around 330 nm. It remains relatively stable when stored in dry, cool conditions, but it can oxidize or isomerize under heat, light, or alkaline pH, necessitating careful handling for research or supplement formulation [35].

### 2.4 Pharmacokinetics and bioavailability

The pharmacokinetic profile of RA reveals extensive first-pass metabolism and relatively modest systemic bioavailability [36]. When administered orally, RA is quickly absorbed; however, only a small proportion of the parent compound reaches circulation intact. Instead, RA is largely metabolized by intestinal enzymes and gut microbiota into diverse phenolic derivatives, including caffeic, ferulic, and *m*-coumaric acids, which themselves possess antioxidant properties [37]. Studies in healthy volunteers show that plasma levels of RA peak within 30 min, primarily in conjugated forms such as glucuronides and sulfates [38]. Most of these metabolites are excreted renally within 24 h, suggesting minimal tissue accumulation and rapid clearance. Despite the low levels of intact RA detected in plasma and tissues, its metabolites likely contribute to the overall biological effects. Routes that circumvent first-pass metabolism, such as intravenous or topical delivery, can increase RA's bioavailability, although these approaches have not been extensively studied in clinical contexts. Nevertheless, RA-containing herbal products have been well-tolerated in humans, and its metabolic byproducts are common dietary phenolics, supporting a favorable safety and clearance profile [38]. Efforts to optimize RA delivery, including formulation with nanocarriers or prodrugs, aim to further enhance its stability and therapeutic efficacy [39].

## 3 Pharmacological profile and mechanisms of action

### 3.1 Antioxidant and free radical scavenging activity

RA exerts robust antioxidant effects that underpin many of its therapeutic benefits [27]. Its polyphenolic structure allows direct quenching of ROS and stabilization of free radicals through resonance, as demonstrated by its strong radical-scavenging activity in assays such as DPPH and ABTS [40]. RA also protects biological membranes by interrupting lipid peroxidation chains and preserving membrane integrity [41]. In models of male reproductive toxicity, RA supplementation significantly reduces markers of oxidative damage, such as malondialdehyde (MDA), while restoring endogenous antioxidants, including glutathione and key enzymes (e.g., superoxide dismutase, catalase, and glutathione peroxidase) [42]. For instance, in doxorubicin-induced testicular injury, RA-treated animals maintain near-normal MDA levels and preserve glutathione peroxidase activity [43]. Mechanistically, RA has been reported to engage the Keap1-Nrf2 antioxidant response axis: in multiple systems it increases Nrf2 nuclear accumulation and upregulates ARE-driven genes (e.g., HO-1, NQO1, GCLC), potentially via both indirect regulation of Keap1 stability (including target deconvolution identifying USP15 as an RA-binding upstream regulator of Keap1) and Keap1-directed mechanisms [[44], [45], [46]]. In the male reproductive system, cell-resolved data remain limited; nevertheless, Nrf2 signaling is demonstrably active in testes, with nuclear Nrf2 accumulation reported in germ and Leydig cells under oxidative stress, and Nrf2 activation protecting Sertoli cells in vitro [47,48]. Additionally, RA can chelate transition metals (Fe^2+^, Cu^2+^) to prevent Fenton-like reactions and intercept reactive nitrogen species, including nitric oxide and peroxynitrite [49]. Through these mechanisms, RA supports mitochondrial function, evidenced by improved ATP levels and membrane potential, and helps maintain sperm motility and DNA integrity under oxidative stress [50].

### 3.2 Anti-inflammatory mechanisms: molecular insights

Chronic inflammation in male reproductive tissues often coincides with oxidative stress, contributing to suboptimal spermatogenesis and reduced fertility [51]. RA exerts notable anti-inflammatory actions by modulating the canonical NF-κB axis and associated upstream signaling, thereby reducing the expression of pro-inflammatory cytokines (TNF-α, IL-1β, IL-6) and enzymes (iNOS, COX-2) [52]. In mechanistic cell-based studies, RA suppresses NF-κB activation at or downstream of IKK-β, attenuating IκBα phosphorylation/degradation and limiting nuclear translocation of p65/p50, with consequent downregulation of NF-κB-dependent transcriptional programs [53,54]. Consistent with this, RA reduces nitric oxide production in activated macrophages by downregulating iNOS, mitigating peroxynitrite formation and inflammatory tissue damage [55]. In testicular inflammation models, RA lowers local pro-inflammatory mediators and normalizes myeloperoxidase activity, indicative of reduced neutrophil infiltration [43]. From a cell-specific perspective, NF-κB activity is present in Sertoli cell nuclei and increases during testicular stress, where it can contribute to germ cell apoptosis; thus, Sertoli cells and resident immune cells represent plausible in-testis targets of RA's NF-κB modulation [56]. However, most reproductive studies quantify pathway readouts in whole-testis lysates, and future work using purified primary testicular cell populations or single-cell approaches is needed to map RA-responsive signaling with cell-type resolution. Collectively, these mechanisms help preserve the immunologically privileged environment of the testes, minimize cellular damage, and complement RA's antioxidant effects.

### 3.3 Endocrine and hormonal effects in reproductive health

**Fig. 2:**
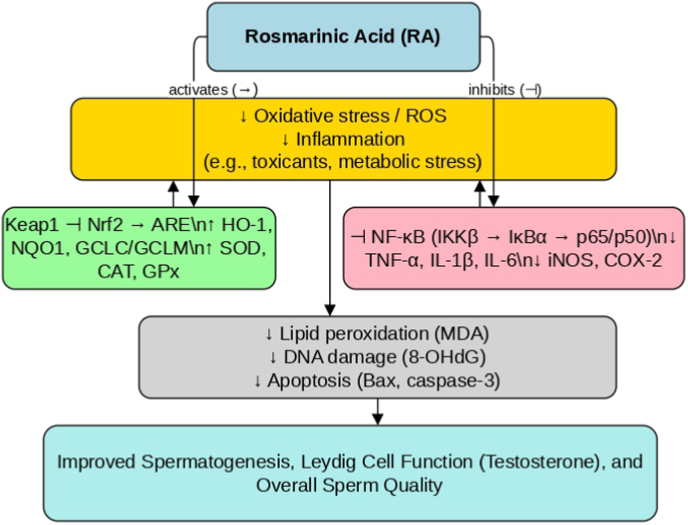
Proposed mechanisms of rosmarinic acid (RA) in male reproductive tissues. Nrf2: Nuclear factor erythroid 2–related factor 2, NF-κB: Nuclear factor kappa-light-chain-enhancer of activated B cells, ROS: Reactive oxygen species.

Oxidative insults and toxic exposures can disrupt testosterone production and dysregulate the hypothalamic–pituitary-gonadal (HPG) axis [57]. In available models, RA appears to preserve endocrine function by protecting steroidogenesis under stress, rather than acting as a direct hormone mimic. For example, in doxorubicin- or PFOA-induced testicular toxicity, RA co-administration attenuates declines in testosterone and, in some studies, helps maintain luteinizing hormone (LH) and follicle-stimulating hormone (FSH) [50]. These patterns are consistent with protection of Leydig cell viability and steroidogenic capacity, likely through mitigation of oxidative damage and apoptosis. RA's anti-inflammatory activity may also contribute indirectly, because systemic inflammatory signaling can suppress gonadotropin release. Importantly, RA does not consistently increase hormone levels in healthy animals, suggesting it is unlikely to disrupt baseline endocrine homeostasis [58]. While the precise molecular mediators in testicular endocrine cells remain incompletely defined, preservation of steroidogenic function under oxidative/inflammatory stress provides a plausible endocrine pathway for RA's reproductive benefits. A schematic overview of these mechanisms across major testicular cell types is provided in Fig. 2.

### 3.4 Neuroprotective and systemic effects on cellular function

Beyond reproductive tissues, RA confers neuroprotection in models of neurodegeneration and cerebral ischemia, partly by crossing the blood–brain barrier and attenuating neuroinflammation and oxidative damage [59]. It has been shown to reduce glial activation and neuronal loss in excitotoxicity models, potentially benefiting reproductive outcomes by maintaining hypothalamic homeostasis [60]. RA's broad antioxidant and anti-inflammatory effects also extend to the cardiovascular system, where it mitigates myocardial damage and improves endothelial function, and to metabolic contexts, where it reduces glycation and oxidative stress in diabetes [61]. Furthermore, RA's immunomodulatory actions include inhibiting abnormal T-cell activation and exerting anti-allergic effects, which could be relevant for individuals with immune-related infertility [44]. Although systemic bioavailability may limit direct testicular targeting, RA's safety profile and multi-organ benefits position it as a viable candidate for long-term use in men with oxidative and inflammatory conditions contributing to infertility. Future strategies to enhance its delivery to reproductive tissues may further optimize these protective effects [62].

## 4 Male infertility: etiology and current therapeutic strategies

### 4.1 Molecular mechanisms of male infertility: oxidative stress, DNA damage, and apoptosis

Male fertility critically depends on balanced redox homeostasis and the maintenance of genomic integrity throughout spermatogenesis [63]. Excessive OS disrupts these processes by attacking sperm membranes, particularly vulnerable due to their high content of polyunsaturated fatty acids, and inducing lipid peroxidation that compromises membrane fluidity and ion channel function [64]. Furthermore, high ROS levels can directly damage sperm DNA through strand breaks and base modifications; since sperm have limited repair capacity, this can result in DNA fragmentation linked to subfertility, higher miscarriage rates, and potential transmission of mutations to offspring [65]. In parallel, OS triggers mitochondrial dysfunction and cytochrome *c* release, activating apoptotic pathways in spermatogenic cells [64]. Conditions such as varicocele and infection often exhibit elevated markers of both OS and apoptosis in germ cells [66]. Additional molecular insults include the overactivation of poly (ADP-ribose) polymerase (PARP), depleting NAD^+^ and ATP, as well as endoplasmic reticulum stress resulting from protein misfolding [64]. These cascades collectively reduce sperm output, impair sperm quality, and compromise fertilization potential. Given the prevalence of oxidative stress in diverse etiologies of male infertility, the notion of “Male Oxidative Stress Infertility (MOSI)” has been proposed to categorize cases characterized by elevated ROS and/or insufficient antioxidant defenses [67]; hereafter, we use MOSI to refer to this oxidative-stress-dominant clinical phenotype**.** Therapeutic interventions aimed at mitigating ROS-induced DNA damage and apoptosis are thus central to preserving sperm functionality.

### 4.2 Endocrine disruptors and environmental factors in male infertility

Growing evidence links environmental exposures and lifestyle choices to declining male reproductive parameters [68]. Endocrine-disrupting chemicals (EDCs), such as phthalates, bisphenol A, polychlorinated biphenyls, and certain pesticides, exert deleterious effects on testicular function, either by generating oxidative stress or by interfering with hormonal pathways essential for spermatogenesis [69]. For instance, PFOA exposure reduces testosterone levels, impairs pituitary hormone release, and causes sperm abnormalities [5]. Heavy metals like cadmium induce ROS-mediated inflammation and apoptosis of Leydig and germ cells [70]. Moreover, environmental heat (e.g., saunas) and radiation (e.g., frequent cell phone use) can exacerbate OS in the testes, leading to lower sperm counts and motility [68]. Lifestyle factors, including cigarette smoking and excessive alcohol intake, further intensify oxidative insults and hormonal disturbances [71]. Obesity also contributes to local heat stress and systemic inflammation, aggravating sperm DNA damage [72]. Alleviating these environmental and lifestyle stressors remains a cornerstone of male infertility management, complemented by antioxidant supplementation to mitigate unavoidable exposures [73]. Nonetheless, regulatory efforts to reduce EDCs in consumer products and the environment are equally crucial to address the public health dimension of male infertility.

### 4.3 Current pharmacological treatments for male infertility: limitations and challenges

Treatment strategies for male infertility often depend on specific diagnoses, but a substantial proportion of cases are idiopathic [74,75]. Hormonal therapies, including human chorionic gonadotropin (hCG) and recombinant FSH, are effective primarily in conditions such as hypogonadotropic hypogonadism. When hyperprolactinemia or ejaculatory dysfunction is present, dopamine agonists and alpha-agonists, respectively, may offer clinical benefit [76]. However, for idiopathic oligozoospermia or asthenozoospermia, standardized pharmacological solutions remain elusive. In such cases, clinicians frequently rely on empirical antioxidant and nutraceutical interventions, given the integral role of oxidative stress in male reproductive dysfunction [77]. Supplements containing vitamins C and E, l-carnitine, coenzyme Q10, zinc, selenium, and various herbs have shown modest improvements in sperm parameters, although definitive evidence for enhanced live birth rates remains limited by heterogeneity in study designs [78]. Off-label use of selective estrogen receptor modulators (e.g., clomiphene) or aromatase inhibitors (e.g., letrozole) aims to optimize the testosterone-to-estrogen ratio in idiopathic infertility, with mixed outcomes. When inflammation or leukocytospermia is identified, anti-inflammatory drugs or antibiotics may be employed, although efficacy can be variable. In immune-mediated infertility, corticosteroids have been tried to reduce anti-sperm antibody titers, albeit with limited success. Ultimately, assisted reproductive techniques (ART), such as intrauterine insemination (IUI) or in vitro fertilization with intracytoplasmic sperm injection (IVF–ICSI), remain the definitive approach for many couples, bypassing several sperm deficiencies [76]. Yet ART procedures do not directly address underlying male health issues and can be expensive or inaccessible. The lack of robust, targeted medications, together with inconsistent results from empiric therapies, underscores the demand for new therapeutic avenues. Research into phytochemicals like rosmarinic acid, which exhibit antioxidant, anti-inflammatory, and endocrine-protective properties with minimal adverse effects, reflects an emerging strategy to enhance male fertility outcomes [51].

### 4.4 Potential positioning of rosmarinic acid across common clinical male infertility phenotypes

Male infertility is clinically heterogeneous, and the translational value of RA likely depends on whether oxidative stress and inflammation are dominant drivers in a given phenotype [79,80]. In idiopathic oligozoospermia and asthenozoospermia, elevated seminal ROS, impaired mitochondrial function, and sperm DNA fragmentation are frequently reported, even when standard semen analysis is only modestly abnormal [[79], [80], [81]]. Because RA improves oxidative balance, mitochondrial indices, and sperm motility in multiple experimental settings (see Table 1 and refs. already cited for preclinical efficacy), it may be most rationally positioned as an adjunct in patients with biochemical evidence of oxidative stress (e.g., elevated lipid peroxidation products, 8-OHdG, or high sperm DNA fragmentation) rather than as a universal empiric supplement for all idiopathic cases [80,81]. In teratozoospermia, where abnormal spermiogenesis can be exacerbated by oxidative injury within the seminiferous epithelium, RA's cytoprotective and anti-apoptotic actions provide a mechanistic rationale; however, direct evidence linking RA to improved morphology in clinically representative models remains limited and should be prioritized in future work [81]. Varicocele-associated infertility is another phenotype where RA may have mechanistic relevance, because varicocele is commonly linked to scrotal hyperthermia, testicular hypoxia, venous stasis/reflux, and secondary oxidative and inflammatory stress within the testis and epididymis [82,83]. In this context, RA could be explored as an adjunct strategy, particularly in men with persistent oxidative stress before or after varicocelectomy, using endpoints that include semen parameters, sperm DNA fragmentation, oxidative stress biomarkers, and inflammatory cytokine profiles [82,83]. Finally, immune/inflammatory infertility (including leukocytospermia and elevated seminal cytokines) represents a setting where RA's NF-κB-linked anti-inflammatory effects could be relevant [84], and where informative translational readouts include seminal leukocyte counts and cytokine panels (e.g., TNF-α, IL-6) alongside semen analysis [85,86]. At the same time, because immune activation can reflect infection or other treatable etiologies, RA should be evaluated as a complementary approach rather than a substitute for etiologic diagnosis and standard-of-care management.

## 5 Experimental evidence: rosmarinic acid in male fertility

### 5.1 Effects of rosmarinic acid on sperm quality and motility (in vitro and in vivo)

A growing number of studies demonstrate that RA supports sperm viability and function under various stress conditions. *In vitro*, RA added to semen extenders has improved sperm parameters in several species, including boars. At concentrations of 40 μM, RA preserved motility, membrane integrity, and acrosomal integrity during extended liquid storage at 17 °C, correlating with enhanced antioxidant enzyme activity and reduced MDA levels [87]. RA was also reported to increase mitochondrial ATP content and stabilize membrane potential, suggesting an energy-regulating function through AMPK activation. Similar benefits were observed for cryopreserved boar sperm, where RA improved post-thaw motility and in vitro fertilization outcomes [87,88]. *In vivo* investigations reinforce RA's protective effects. In a rodent model of metronidazole (MTZ)-induced subfertility, RA (5 or 15 mg/kg) prevented significant reductions in sperm count, motility, and morphology. Histological analyses showed preserved seminiferous tubule architecture and reduced germ cell degeneration compared to untreated controls [89]. RA also alleviated oxidative damage in rat models of testicular ischemia–reperfusion injury, supporting higher sperm quality and reduced apoptotic cell death [51]. These findings collectively indicate that RA enhances sperm resilience by reducing ROS-mediated damage and sustaining energy metabolism, without adversely affecting normal sperm parameters. For a concise overview of representative preclinical studies on RA's role in male fertility, see Table 2.

**Table 2:** Representative preclinical studies on rosmarinic acid (RA) in male fertility, including experimental cycle, oxidative stress/inflammatory readouts (values where reported), and study limitations. DOX, doxorubicin; CP, cyclophosphamide; i.p., intraperitoneal; MTZ, metronidazole; PFOA, perfluorooctanoic acid; STZ, streptozotocin; TAC, total antioxidant capacity; PC, protein carbonyl. †HED calculation (BSA method): HED (mg/kg) = animal dose (mg/kg) × (Km_animal/Km_human), using Km_mouse = 3, Km_rat = 6, Km_human = 37; mg/day assumes 60-kg adult.

Model (species)	Trigger/phenotype	Experimental cycle (total; key timing)	RA regimen (route; schedule)	Dose (animal)	HED†	Key reproductive endpoints	Key oxidative/inflammatory readouts (values if reported)	Study limitations (concise)	Ref
Metronidazole-induced reversible infertility (rat)	Metronidazole (MTZ) exposure	∼4 weeks; endpoints reported ∼30 days post-start	RA pre-treatment before MTZ (i.p.; timing reported)	5 or 15 mg/kg	5 mg/kg → ∼49 mg/day; 15 mg/kg → ∼146 mg/day	Sperm count/motility/morphology; testis ultrastructure	OS/inflammation biomarkers not consistently reported for this model (NR)	Short-term; limited mechanistic profiling; no mating/pregnancy endpoints	[90]
Diabetes-related testicular injury (mouse)	STZ-induced diabetes	STZ induction (5 days) + RA treatment 7 weeks	RA (i.p.; 6 × /week for 7 weeks)	5 or 15 mg/kg	5 mg/kg → ∼24 mg/day; 15 mg/kg → ∼73 mg/day	Sperm quality; histology; apoptosis markers	Redox markers assessed (e.g., lipid peroxidation, GSH, SOD/CAT) but values primarily presented graphically across assays (NR)	Primarily biochemical endpoints; limited fertility endpoints; dose-response mainly preclinical	[51]
Environmental toxicant-related injury (rat)	PFOA exposure	14 days total	RA (oral gavage; 14 days)	40 mg/kg	∼389 mg/day	Sperm count/motility/morphology; testosterone/LH/FSH; organ weights	OS/inflammation markers not primary endpoints (NR)	Short duration; exposure model-specific; limited mechanistic depth	[50]
Chemotherapy-related gonadotoxicity (rat)	Doxorubicin (DOX)	∼10–11 days; DOX on day ∼7, sacrifice ∼ day 11	RA (oral; alongside DOX protocol)	75 mg/kg/day	∼730 mg/day	Histopathology; sperm indices; endocrine endpoints	Reported oxidative/inflammatory readouts include MDA and TNF-α (values NR in summary table)	Limited dose exploration; short follow-up; fertility endpoints typically absent	[91]
Chemotherapy-related gonadotoxicity (rat)	Cyclophosphamide (CP)	14 days total; CP administered during last 7 days	RA (i.p.; daily × 14 days; co-treatment with CP last 7 days)	20 mg/kg/day	∼195 mg/day	Testis structure; apoptosis markers	**Serum MDA:** CP 2.5 ± 0.9 vs RA + CP 1.7 ± 0.03 (nmol/mL); Bax downregulated with RA co-treatment	Primary focus on apoptosis/oxidative marker(s); limited semen functional endpoints; short duration	[92]
Electromagnetic radiation testicular toxicity (rat)	Cell phone (915 MHz) or UHF (2450 MHz) exposure	30 days; radiation 60 min/day; RA given 1 h prior daily	RA (oral gavage; daily × 30 days)	20 mg/kg/day	∼195 mg/day	Histology (Sertoli/germ cell indices); seminiferous metrics	Approx. from reported plots: **MDA (nmol/mg protein)** Control ∼3.5, UHF ∼7.3, RA + UHF ∼5.8; **NO (nmol/mg protein)** Control ∼15, UHF ∼37, RA + UHF ∼31; **TAC (μg/mg protein)** Control ∼1.5, UHF ∼0.7, RA + UHF ∼1.0; plus SOD/CAT/GPx/GSH/PC trends consistent with antioxidant protection	Values partly graph-based; not fertility/mating outcomes; exposure paradigm may not generalize to clinical infertility	[42]
Sperm preservation model (boar semen, ex vivo)	Liquid storage stress (17 °C)	Storage duration per extender protocol	RA added to extender	40 μM	—	Motility; membrane integrity; mitochondrial status	Lower MDA and improved antioxidant enzyme activity reported during storage (values NR in summary table)	Ex vivo model; species-specific; not directly predictive of human infertility treatment	[87]

### 5.2 Dose-route considerations and translational dose framing

Across the reproductive models summarized in Table 1, protective effects are most consistently reported in the low-to-mid mg/kg range, particularly with intraperitoneal dosing (commonly 5-15 mg/kg), whereas higher doses are often used when RA is administered orally (e.g., 40 mg/kg). This route dependence is biologically plausible given RA's extensive first-pass metabolism and modest oral bioavailability. When translated using standard body-surface-area scaling, commonly effective rodent doses map to an approximate RA-equivalent intake in the tens to low hundreds of milligrams per day for a 60-kg adult, while higher oral preclinical regimens map closer to the high hundreds of milligrams per day. These estimates should be considered hypothesis-generating and underscore the need for future fertility-targeted trials to incorporate dose-ranging, pharmacokinetic readouts, and semen oxidative stress biomarkers (e.g., lipid peroxidation products, 8-OHdG, sperm DNA fragmentation) alongside conventional semen analysis.

### 5.3 Protective effects of rosmarinic acid against testicular oxidative stress and apoptosis

Oxidative injury to testicular tissue is a key driver of male infertility in various pathological conditions. RA has consistently shown the capacity to mitigate this damage. In a doxorubicin model of chemotherapy-induced gonadotoxicity, co-administration of RA (10 days, intragastric) preserved seminiferous tubule integrity, decreased germ cell apoptosis, and maintained antioxidant enzyme activity in the testes [90]. Notably, RA prevented the sharp rise in MDA levels and safeguarded Leydig cell function, reflected by significantly higher testosterone levels in RA-treated animals. Similarly, RA has demonstrated protective effects in diabetic models. Chronic hyperglycemia often results in testicular OS and reduced sperm parameters; however, RA administration in streptozotocin-induced diabetic mice preserved sperm count and motility and restored antioxidant capacity in testicular tissue [51]. Environmental toxicants also induce testicular oxidative damage: in PFOA-exposed rats, RA (40 mg/kg) prevented testicular atrophy, oligospermia, and aberrant sperm morphology through its ROS-scavenging properties [50]. Common to these diverse models is RA's ability to stabilize redox balance, inhibit apoptotic pathways (e.g., Bax/Bcl-2 ratio, caspase-3 activation), and maintain tissue architecture, thereby promoting fertility outcomes.

### 5.4 Animal models of male infertility: role of phytochemicals in sperm protection

Animal studies have highlighted the broad capacity of phytochemicals, quercetin, resveratrol, epigallocatechin gallate, and others, to defend sperm and testicular cells against specific insults [93]. RA is particularly noteworthy for combining antioxidant, anti-inflammatory, and metabolic regulatory actions at relatively low doses. In many experimental designs, RA administered after the onset of injury (e.g., chemotherapy, diabetes, toxicant exposure) still facilitated recovery of fertility markers, suggesting a therapeutic window beyond mere prophylaxis. RA has also been proposed as a candidate for combination therapies, potentially synergizing with other antioxidants or metabolic enhancers like vitamin E or coenzyme Q10 [50,94]. Toxicological assessments indicate that RA is generally safe at doses that confer reproductive benefits, further supporting its clinical promise.

### 5.5 Hormonal modulation by rosmarinic acid: testosterone and reproductive function

Leydig cell damage and suppressed gonadotropins are common features of oxidative and inflammatory stress in male infertility. RA's protective actions frequently involve preserving normal hormone levels rather than artificially elevating them. In PFOA-exposed rats, for instance, RA prevented the decline in testosterone, LH, and FSH, indicating both testicular and pituitary protection [50]. Similar patterns have been reported in doxorubicin-treated rodents, where RA co-treatment maintained significantly higher testosterone compared to untreated controls [90]. Because RA does not raise hormone levels in healthy animals, it is unlikely to disrupt endocrine homeostasis. Instead, by reducing local inflammation and oxidative damage, RA supports Leydig cell integrity and steroidogenic capacity, thereby sustaining the hormonal milieu essential for fertility.

## 6 Safety, toxicity, and clinical relevance

### 6.1 Toxicological evaluation of rosmarinic acid: dosage and long-term safety

RA is generally regarded as a safe phytochemical, in part because it is naturally abundant in culinary herbs and has a long history of dietary exposure. Acute and subchronic animal toxicology studies generally report good tolerability across a wide dose range, with no consistent signals of organ toxicity in routine hematology or clinical chemistry panels. In reproductive toxicity-focused experiments, RA administered at low-to-moderate doses (commonly 5-15 mg/kg in rodents) does not appear to disrupt testicular histology, spermatogenic architecture, or baseline sperm indices, aligning with its antioxidant profile [16,95]. Human evidence remains limited and is largely derived from RA-rich botanical preparations rather than purified RA. Nevertheless, controlled studies evaluating RA-rich extracts have not identified treatment-related safety concerns at daily RA-equivalent intakes in the low hundreds of milligrams [38,96]. Importantly, these human studies have not been designed to evaluate semen parameters, reproductive hormones, or fertility outcomes; therefore, current clinical evidence should be interpreted primarily as preliminary safety/PK support rather than proof of reproductive efficacy. To contextualize potential risks, a human PK trial reported a maximum total serum RA concentration of ∼162 nmol/L after a single 500 mg oral RA dose (≈0.16 μM), which is substantially below the micromolar concentrations typically used in mechanistic in vitro studies [38]. Reported pro-oxidant behavior has mainly occurred in vitro under transition metal–rich conditions (e.g., Cu/Fe-dependent redox cycling) and at higher experimental concentrations, which may not reflect physiological exposure [97]. Likewise, antiplatelet/anti-adhesive effects have been observed ex vivo/in vitro at **∼**10-90 μM RA, again well above typical circulating levels after oral intake, yet caution remains prudent in individuals receiving antiplatelet/anticoagulant therapy or those using concentrated herbal products [98,99]. Because RA can interact with drug-metabolizing enzymes (CYP/UGT) in vitro, early-phase clinical studies in infertility populations should include medication reconciliation and basic safety/bleeding surveillance where applicable [100].

### 6.2 Clinical evidence of rosmarinic acid and RA-rich extracts: what human studies do and do not show

Although fertility-targeted clinical trials are currently lacking, several human studies provide useful signals regarding RA exposure, pharmacokinetics, and short-to mid-term tolerability. In a randomized controlled pharmacokinetic study in healthy volunteers, single oral administration of *Melissa officinalis* extract delivering up to 500 mg RA-equivalent was not associated with clinically meaningful changes in routine safety laboratories, and no treatment-related adverse events were reported; RA exposure peaked within hours and was observed predominantly as conjugated metabolites [38]. Beyond pharmacokinetics, small controlled studies of RA-rich preparations have reported biological activity in non-reproductive contexts (e.g., allergic or inflammatory readouts), supporting systemic bioactivity at oral doses in the 50–200 mg/day range, albeit with endpoints unrelated to male fertility [96]. Longer-duration exposure has been examined in placebo-controlled trials of RA-rich *Melissa officinalis* extract at 500 mg RA/day, including a 24-week study in mild Alzheimer's disease and a 96-week study in older adults with subjective or mild cognitive impairment; both trials primarily support tolerability and do not report reproductive endpoints [101,102]. Collectively, available human data suggest that RA-rich preparations can achieve measurable systemic exposure and appear tolerable in the short to midterm; however, they do not resolve the central translational question for this review, namely whether RA improves semen quality, sperm DNA integrity, or pregnancy outcomes. Notably, an in vitro human sperm study reported impaired sperm function at high micromolar RA concentrations, reinforcing the need for careful dose-ranging and fertility-specific safety monitoring in future clinical trials [103].

### 6.3 Pharmacokinetics and drug interactions of rosmarinic acid in clinical settings

RA undergoes rapid absorption but extensive first-pass metabolism, resulting in low systemic bioavailability of the parent compound [96]. Phase II conjugation pathways (glucuronidation, sulfonation) and gut microbial metabolism yield phenolic derivatives such as caffeic and ferulic acids, which themselves possess antioxidant properties. These metabolites are typically excreted renally within 24 h, reducing the likelihood of accumulation or significant toxicity.

To date, no major clinical drug interactions have been reported. Although RA might transiently share metabolic pathways (e.g., UDP-glucuronosyltransferases) with certain medications, its short plasma half-life and moderate concentrations likely preclude substantial competitive inhibition [100]. RA's potential to modulate gut microbiota may alter its own metabolism or that of concurrently administered compounds, but no harmful effects have been documented [96,104]. When combined with other antioxidants or anti-inflammatory agents, RA may exhibit additive benefits; excessive suppression of inflammatory pathways, however, remains a theoretical concern rather than an established clinical issue [105,106].

### 6.4 Regulatory aspects and clinical guidelines for phytochemical use in male fertility

No official guidelines currently endorse rosmarinic acid as a standalone treatment for male infertility. Professional associations, such as the American Urological Association and the European Association of Urology, acknowledge antioxidants as adjunctive options but do not specify RA in their recommendations [107]. Within the United States, RA-containing products often fall under the Dietary Supplement Health and Education Act (DSHEA), permitting structure/function claims but prohibiting explicit disease-treatment claims unless proven through rigorous clinical trials. European authorities similarly allow RA-rich herb extracts as food additives or supplements, given their long history of safe consumption. For RA to be formally integrated into infertility management, robust clinical evidence, ideally from randomized controlled trials, would be required. Nonetheless, many clinicians already advise antioxidant supplementation, including RA-rich herbal extracts, for idiopathic cases characterized by oxidative stress. Standardization of RA content in such products, alongside confirmation of safety and efficacy in reproductive-age men, will be critical for future guidelines. In the interim, RA remains a promising, albeit not yet officially sanctioned, tool for mitigating oxidative damage and preserving testicular function in men seeking fertility support [103].

## 7 Formulation and delivery strategies

### 7.1 Nanotechnology-based drug delivery systems for rosmarinic acid

Achieving optimal tissue delivery and bioavailability for RA remains a key challenge [36]. Nanotechnology-based carriers offer promising solutions by protecting RA from rapid metabolism, enhancing its solubility, and enabling controlled release and (in principle) targeted delivery to reproductive tissues. Polymeric nanoparticles, commonly made from biocompatible polymers such as poly (lactic-co-glycolic acid) (PLGA), can encapsulate RA and preserve its antioxidant activity while supporting sustained release [108]. Similar approaches include solid lipid nanoparticles and liposomes, which may further improve chemical stability and cellular uptake [109]. Surface functionalization could, in the longer term, be leveraged for site-directed delivery (e.g., *trans*-blood–testis barrier transport), although this remains a proof-of-concept extrapolated from other bioactive compounds [110]. Importantly, in vivo studies in non-reproductive indications suggest that RA nano formulations can increase tissue exposure relative to free RA, supporting the feasibility of formulation-driven PK optimization [111]. For male infertility, however, systematic intratesticular PK studies are still lacking, and future work should explicitly quantify testicular distribution, metabolism, and on-target engagement after nano formulation.

### 7.2 Enhancing bioavailability of polyphenols: strategies and challenges

Like many polyphenols, RA exhibits strong activity in vitro yet limited efficacy in vivo due to extensive first-pass metabolism and low plasma residence times [112]. Several formulation strategies are under investigation (Table 3):

• **Nanoparticle encapsulation**: Protects RA from rapid degradation and enhances intestinal absorption.
• **Prodrug development**: Lipophilic modifications that improve membrane permeability and subsequent intracellular release of active RA.
• **Complex formation**: Complexing RA with cyclodextrins, phospholipids, or other carriers (e.g., phytosomes) to boost solubility and stability.
• **Metabolism inhibition**: Co-administration of agents like piperine may slow glucuronidation, prolonging RA's half-life [115].
• **Alternative administration routes**: Sublingual, buccal, and intranasal delivery bypass first-pass metabolism; transdermal methods may improve localized tissue exposure.

**Table 3:** Strategies to improve rosmarinic acid (RA) bioavailability and translational considerations. This table summarizes formulation and chemical approaches used to enhance RA exposure, highlighting their principal advantages, practical limitations (including manufacturing, stability, biodistribution, and drug–drug interaction risk), and the current level of supporting evidence. Abbreviations: PLGA, poly (lactic-co-glycolic acid); BTB, blood–testis barrier; PK, pharmacokinetics; DDI, drug-drug interaction; CYP, cytochrome P450; UGT, uridine diphosphate-glucuronosyltransferase.

Strategy	Representative approaches	Key advantages	Key limitations/translation notes	Evidence level
Nanocarriers (polymeric/lipid)	PLGA nanoparticles; solid lipid nanoparticles; liposomes	Protect RA from degradation; improve solubility; controlled/sustained release; potential for tissue targeting	Scale-up and batch reproducibility; long-term stability; biodistribution and immunogenicity assessment; testis delivery requires dedicated PK and BTB-penetration studies	Strong in vitro; limited in vivo for RA; minimal fertility-specific PK [108,109,111]
Prodrug/chemical modification	Short-chain (≤C4) RA esters; structure-guided esterification	Increased permeability and systemic exposure; may outperform parent RA in exposure/potency	New chemical entity; requires validated in vivo cleavage to active RA; chain-length dependent toxicity risk; more demanding regulatory path	In vivo (rat) PK + in vitro potency shown [113]
Complexation/phytosome	Cyclodextrin inclusion complexes; phospholipid complexes (phytosomes)	Relatively simple manufacturing; improved apparent solubility; generally favorable tolerability	Exposure gains may be modest; may not overcome rapid phase II conjugation; product-dependent reproducibility	Moderate (supported across polyphenols; limited RA fertility data) [114]
Metabolism modulation (bioenhancers)	Piperine co-administration; inhibitors of glucuronidation/efflux (conceptual)	Can increase exposure by reducing conjugation/efflux	Drug–drug interaction (DDI) risk via CYP/UGT and transporters; variable effects; not ideal in polypharmacy	Preclinical supportive; requires DDI monitoring [115]
Alternative delivery routes	Buccal/sublingual; intranasal; transdermal	Bypass first-pass metabolism; may improve systemic exposure; potential localized delivery	Formulation complexity; mucosal/skin tolerability; dose limitations; fertility-specific validation lacking	Early-stage; fertility translation uncertain [116]

Although these approaches hold promise, their translational profiles differ substantially. Nanocarriers (polymeric, lipid, or hybrid systems) have the strongest body of formulation and in vitro release/uptake data for RA, but clinical translation requires scalable manufacturing, long-term stability, reproducible loading/release, and careful evaluation of immunogenicity and biodistribution, particularly if testis targeting is intended [[108], [109], [110], [111]]. Prodrug strategies can improve permeability and systemic exposure; for example, short-chain (≤C4) esterification markedly increased oral bioavailability in rats, whereas longer-chain esters introduced cytotoxicity, highlighting that chemical modification must be tightly optimized [113]. Complexation approaches (e.g., cyclodextrins, phospholipid complexes/phytosomes) are typically simpler to manufacture and may be attractive for early clinical testing, but they often provide more modest exposure gains and may not address rapid phase II conjugation. Finally, co-administration with metabolism modulators (e.g., piperine) and alternative routes (sublingual/buccal/intranasal) can raise exposure but introduce higher interaction and tolerability uncertainty, warranting a conservative risk-benefit assessment in fertility patients who may be using concurrent medications [114,115]. Overall, we view standardized oral formulations and simple complexation as the most near-term clinically tractable options, whereas targeted nanocarriers and prodrugs are promising but require dedicated reproductive PK/toxicology packages before translation.

### 7.3 Oral versus topical administration of phytochemicals for reproductive health

For male infertility, oral administration is the most practical route, supported by preclinical studies demonstrating RA's systemic absorption and testicular benefits [89]. Although first-pass metabolism diminishes intact RA levels, animal models confirm that sufficient amounts, or metabolites with similar activities, can reach the testes. Oral dosing is thus likely to be the mainstay if RA advances to clinical trials for male fertility. Topical application has garnered interest but is less straightforward. While scrotal skin is relatively permeable (hence its use for transdermal testosterone), reliable RA penetration into deeper testicular tissues is uncertain [116]. Formulating RA into gels or patches with penetration enhancers could theoretically achieve higher local concentrations and limit systemic exposure, but clinical validation is lacking. Another potential application is direct inclusion of RA in semen-processing media to mitigate oxidative stress during in vitro fertilization procedures, an approach successful in livestock studies [87]. In summary, oral administration remains the leading option for RA-based interventions, balancing patient compliance with demonstrated efficacy. Topical or localized delivery could be explored in specialized scenarios but requires further formulation research. Continued advances in nanoparticle technology and bioavailability enhancement, coupled with evidence from animal and early clinical studies, will guide the optimal administration strategy for RA in male infertility management.

## 8 Knowledge gaps and future directions

### 8.1 A critical review of knowledge gaps

Despite robust preclinical evidence supporting the fertility-enhancing potential of RA, several gaps remain. Clinical research gaps: human infertility-focused data are lacking; no randomized trials have evaluated RA supplementation in men with infertility or subfertility. Consequently, optimal dosing, treatment duration, and patient selection (including men with MOSI) remain speculative [44]. Trials should also prioritize clinically meaningful outcomes, including sperm DNA fragmentation, pregnancy rates, and live birth, alongside conventional semen analysis.

Mechanistic research gaps: while RA's antioxidant and anti-inflammatory activities are well documented, its testis- and sperm-specific molecular targets remain incompletely characterized [117]. In particular, whether RA activates Nrf2 and/or suppresses NF-κB in human testicular cell types has not been definitively demonstrated, and pathway readouts are often derived from whole-tissue lysates rather than cell-resolved analyses [118]. RA-induced AMPK activation observed in boar sperm suggests a potential role in energy metabolism, but validation in human sperm and testicular cells is needed [119]. Pharmacokinetic gaps: many animal studies rely on non-oral routes (e.g., i.p.), raising questions about whether oral RA achieves sufficient intratesticular exposure to reproduce these effects. Rigorous reproductive pharmacokinetic studies should quantify testicular distribution, metabolite profiles, and exposure–response relationships after clinically feasible oral dosing [89]. Formulation-development gaps: while several bioavailability-enhancing strategies are proposed, few have been evaluated with fertility-relevant endpoints or with standardized manufacturing and stability packages. Head-to-head comparisons that integrate PK, testicular biodistribution, and safety are needed to identify the most clinically tractable formulation approaches. Finally, broader etiologic coverage remains incomplete; beyond chemically or physically induced oxidative stress models, RA's potential role across common clinical infertility categories requires targeted investigation.

### 8.2 Prospects for clinical trials: can rosmarinic acid be a future therapeutic agent?

The strong preclinical rationale for RA's use in male infertility supports the need for well-designed clinical trials. An initial strategy could involve a pilot study in men with idiopathic infertility consistent with MOSI (e.g., elevated seminal ROS and/or impaired antioxidant capacity), randomizing participants to RA supplementation or placebo over one or two spermatogenic cycles [50]. Key endpoints would include changes in semen parameters, sperm DNA fragmentation, and markers of oxidative stress, followed by larger trials assessing pregnancy and live birth rates. Additional cohorts could include patients undergoing chemotherapy or with occupational toxin exposure, where RA's antioxidant effects may preserve testicular function. Given RA's generally favorable safety profile, it is also feasible to explore it as an adjunct therapy. For example, RA co-administration with clomiphene citrate might yield synergistic improvements in sperm quality, or RA supplementation post-varicocelectomy could enhance testicular recovery [51]. Ultimately, randomized controlled trials that demonstrate meaningful clinical outcomes, particularly improved pregnancy rates, would be required for RA to gain acceptance as a targeted, evidence-based treatment for male infertility.

### 8.3 Combination therapies for male infertility: the role of phytochemicals

Because male infertility often has multifactorial origins, combining RA with other interventions may offer enhanced benefits [103]. Empirical antioxidant “cocktails” are already commonplace, but rationalizing these combinations based on distinct mechanisms could improve efficacy. RA's strong antioxidant and anti-inflammatory properties could be paired with agents that bolster mitochondrial function (e.g., l-carnitine, coenzyme Q10) or those providing key micronutrients (e.g., zinc, folate) to reinforce sperm DNA integrity. Meanwhile, integrating RA with hormonal treatments, such as clomiphene, may protect newly produced sperm from oxidative damage.

Future research should focus on evaluating whether such combinations offer true synergy, or if benefits plateau. Overlapping metabolic pathways among multiple polyphenols might create redundancy or even reduce individual compound bioavailability. Nevertheless, RA's track record in preclinical models suggests it could serve as a pivotal component of multifaceted regimens aimed at safeguarding male reproductive health.

## 9 Conclusion

Male infertility has a profound global impact, with oxidative stress identified as a key mechanistic driver across diverse etiologies. RA emerges from this review as a potent natural compound capable of countering oxidative and inflammatory damage in the male reproductive system. Preclinical models consistently demonstrate RA's ability to preserve sperm quality, maintain spermatogenesis, and protect steroidogenic function under harmful conditions ranging from toxicant exposure to metabolic disturbances. These findings, coupled with RA's favorable safety profile, highlight its promise as a potential therapeutic or adjunct for improving male fertility. Despite these advances, translating RA's laboratory success into clinical practice will require rigorous human trials. Determining effective dosages, treatment durations, and patient selection criteria, particularly in men with documented oxidative stress, remains a priority. Moreover, addressing formulation challenges to enhance RA's bioavailability will be critical for achieving consistent systemic and testicular levels. If these hurdles are overcome, RA could complement or even reduce the need for invasive assisted reproductive technologies, offering a practical, mechanism-driven strategy to mitigate infertility. In a landscape where existing treatments frequently yield heterogeneous outcomes, rosmarinic acid exemplifies a scientifically grounded, well-tolerated phytochemical that could fill an unmet therapeutic niche. Its multifaceted actions against oxidative stress, inflammation, and cellular injury underscore its potential role in both preventative and restorative contexts. With appropriately designed clinical studies, RA may soon join the arsenal of evidence-based interventions aimed at safeguarding and enhancing male reproductive health.

## Funding

This work was supported by the 10.13039/501100021077Vice Chancellor for Research and Technology of 10.13039/501100004320Shiraz University of Medical Sciences, Shiraz, Iran [grant number 34823].

## CRediT authorship contribution statement

**Morteza Jafarinia:** Investigation, Methodology, Writing – review & editing. **Sepide Goharitaban:** Investigation, Methodology, Software, Writing – original draft, Writing – review & editing. **Bahia Namavar Jahromi:** Writing – original draft, Writing – review & editing. **Mohammad Ebrahim Parsanezhad:** Conceptualization, Supervision, Writing – review & editing. **Sina Vakili:** Methodology, Project administration, Supervision, Writing – original draft, Writing – review & editing.

## Declaration of competing interest

All authors confirm that there are **no financial, personal, academic, or institutional relationships** that could be perceived as influencing the integrity, objectivity, or interpretation of the work presented in this manuscript.

All authors have reviewed and approved this declaration.

## Data Availability

No data was used for the research described in the article.
